# Microstructure, Mechanical Properties and Wear Behaviors of Ultrafine-Grain WC-Based Cermets with Different Binder Phases Fabricated by Spark Plasma Sintering

**DOI:** 10.3390/ma17030659

**Published:** 2024-01-29

**Authors:** Kangwei Xu, Zhe Wang, Peipei Cao, Xiangyang Peng, Chao Chen, Qingsong Liu, Shufeng Xie, Xiaoyu Wu, Yongxin Jian

**Affiliations:** 1 State Key Laboratory for Marine Corrosion and Protection, Luoyang Ship Material Research Institute, Luoyang 471023, China; xukangwei1989@163.com (K.X.);; 2 School of Materials Science and Engineering, Zhengzhou University, Zhengzhou 450001, China; 3 China Nuclear Power Technology Research Institute Co., Ltd., Shenzhen 518000, China; 4 School of Mechanical Engineering, Xi’an Jiaotong University, Xi’an 710049, China

**Keywords:** SPSed ultrafine-grain WC-based cermet, HEA binder phase, microstructure, mechanical properties, wear properties

## Abstract

In this work, to explore potential substitutions for the Co binder phase, ultrafine-grain WC-based cermets with various binder phases of Co, Ni and AlCoCrNiFeCu HEA were prepared using the SPS method. Based on SPS, WC-based cermets were fabricated at higher speed, showing fine carbide particles less than 410 μm. The microstructure, mechanical properties and wear properties were systematically evaluated. By comparison, the grain size of WC was the lowest for WC-10Co, while WC-10 HEA cermet held the coarsest WC particles. The hardness and fracture toughness of WC-10 HEA were the best among all three samples, with values of 93.2 HRA and 11.3 MP·m^1/2^. However, the bending strength of WC-10HEA was about 56.1% lower than that of WC-10Co, with a value of 1349.6 MPa. The reduction in bending strength is attributed to the lower density, formation of a newly Cr-Al rich phase and coarser WC grains. In dry sliding wear conditions, WC-10 HEA showed the lowest wear rate (0.98 × 10^−6^ mm^3^/(N·m)) and coefficient of friction (0.19), indicating the best wear resistance performance. This reveals that WC-based cermet with a HEA binder phase has superior wear performance due to the higher hardness and good self-lubricating effect of the wear products.

## 1. Introduction

WC-based cermets have wide applications in the fields of machining, metallurgy and mining owing to their high hardness, good strength and superior wear resistance [1,2,3]. Until now, WC-based cermets with Co as the binder phase were the most common in industrial applications. This is mainly because Co has excellent wettability to WC so as to guarantee the mechanical properties [4,5]. However, the corrosion and oxidation resistance of WC-Co cermets are not satisfactory [6,7], which restricts their applications in many hazardous environments. On the other hand, Co is a type of rare metal and is a strategic resource [8,9,10]. Reducing the usage of Co or developing substitute elements has been always a hot topic among materials researchers [2,7,11,12,13,14]. In this case, it is of great worth to explore alternative binder phases to replace the traditional Co in WC-based cermet.

To date, a few studies have been conducted to explore potential substitutes for the Co binder phase in WC-based cermets [15]. Su et al. [16] reported that Ni addition decreased WC grain size so that the hardness and transverse rupture strength could be improved. In addition, the corrosion rate also decreased by about 50%, confirming the potential to substitute Co by Ni. Furthermore, Rong et al. [9] prepared ultrafine WC-Ni cemented carbides by spark plasma sintering with VC and TaC as WC grain growth inhibitors. They found that the WC-Ni cemented carbide showed higher hardness than WC-Co cemented carbides, while the bending strength also possessed very high values. Santos et al. [7] investigated the microstructure and corrosion resistance of WC-based cermet with a 10% Ni-Cr-Mo binder phase and confirmed its superior corrosion resistance to acidic chloride and sulphate. On the other hand, the effects of Cu on the microstructure, mechanical properties and wear resistance of WC-Co cemented carbides have also been investigated [17]. The results proved that Cu addition was beneficial in increasing the hardness and wear resistance of WC-6Co, despite the fact that the density and fracture toughness decreased correspondingly. Above all, it can be concluded that it is feasible to replace the initial Co binder phase in WC-based cermet by other metals, and certain properties can be modified in some degree by a variation in binder phase [18].

In recent years, high-entropy alloy (HEA) has been extensively investigated due to its good overall properties, including mechanical properties, wear resistance and corrosion resistance [19,20]. Thus, the overall properties of WC-based cermets are expected to be further improved by using the binder phase of HEA [21,22]. Zhou et al. [8] compared the microstructure, mechanical properties and corrosion resistance of WC-Co and WC-HEA cemented carbides. Based on their results, WC-HEA samples showed smaller particle size and better corrosion resistance except for higher hardness and strength. A few other research studies on WC-cemented carbides were conducted in succession. Luo et al. fabricated WC-AlCoCrCuFeNi high-entropy alloy composites using spark plasma sintering and characterized the microstructure and mechanical properties. They found that HEA binder showed the advantage of inhibiting the growth of WC grain so as to refine the particle size. Consequently, the hardness and fracture toughness of WC-10HEA are higher than those of commercial WC-Co composites. Additionally, Zhao et al. [2] investigated the microstructure and properties of coarse WC-10CoCrFeMnNi cemented carbide using mechanical alloying and hot-pressing sintering. They found WC-HEA cemented carbide showed better combined properties including hardness, fracture toughness, transverse rupture strength and wear resistance. However, Mueller-Grunz et al. [23] obtained different results for WC-(Al)CoCrCuFeNi cemented carbides prepared with the vacuum sintering method. According to their results, HEA with Cr element was not suitable to be used as binder phase to produce WC-HEA cermets with superior mechanical properties. Above all, most of the current research has demonstrated that HEAs have great potential as the binder phase of WC-based cermets. However, some researchers found opposing results that WC-based cermet with a HEA binder phase showed worse mechanical properties. There is a lack of comparative studies to evaluate the real effect of the HEA binder phase. In addition, the present studies are generally focused on the effects of binder content or varying certain elements on the microstructure and mechanical properties of WC-based cermet [22,24,25]. Thus, it is essential to systematically investigate the effect of the HEA binder phase on the microstructure and comprehensive properties of WC-based cermets compared to most common Co and Ni binder phases.

Compared to traditional sintering techniques, spark plasma sintering (SPS) has the advantage of rapid heating and cooling during the sinter process, so that the WC grains cannot grow during the consolidation process. Consequently, the WC-based cermets generally showed higher strength and hardness owing to the refinement of the WC grain [17,26,27,28]. Thus, the wear resistance is also supposed to be affected by the evolutions of microstructure and mechanical properties [29]. A few studies have demonstrated that the SPS technique is applicable to fabricate ceramics or cermets with superior properties [28,30]. On the other hand, the SPS method can help shorten the sintering time so as to improve the sintering efficiency, which makes it an effective way to prepare ceramics, metals and cermets.

In this work, ultra-fine grain WC-based cermets with 10% binder of Co, Ni and AlCoCrNiFeCu HEA were prepared using the spark plasma sintering method (SPS), and the microstructure and mechanical properties were comparatively studied to indicate the effect of the new HEA binder phase. Furthermore, the wear properties, which closely relate to the lifetime of the machining cutter, were also investigated. By comparing the microstructure and properties, this paper aims to provide good guidelines for designing WC-based cermet with superior anti-wear properties by adjusting the binder phase.

## 2. Materials and Experimental Methods

### 2.1. Raw Powders

**Figure 1:**
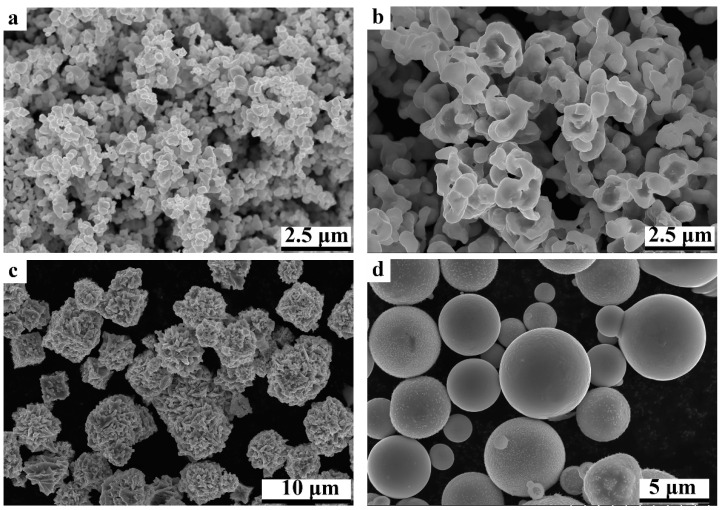
Raw powders of WC, Co, Ni and HEA: (**a**) WC; (**b**) Co; (**c**) Ni; (**d**) HEA.

Commercial WC, Co, Ni and AlCoCrNiFeCu HEA powders were used to prepare the WC-based cermets in this work. The specific information on the raw powders is listed in Table 1. All the raw powders have a purity of more than 99.9%. The average diameter of the WC powder is 0.6 μm, while those of Co and Ni are 1.0 μm, respectively, but the average diameter of AlCoCrFeNiCu powder is 10 μm. Figure 1 shows the morphology of the different raw powders. As shown, WC powders are fine particles with irregular shapes. Co and Ni powders also show irregular particles, but obvious agglomeration can be observed. AlCoCrNiFeCu HEA powders are spherical, but the diameters are obviously larger than Co and Ni.

**Figure 2:**
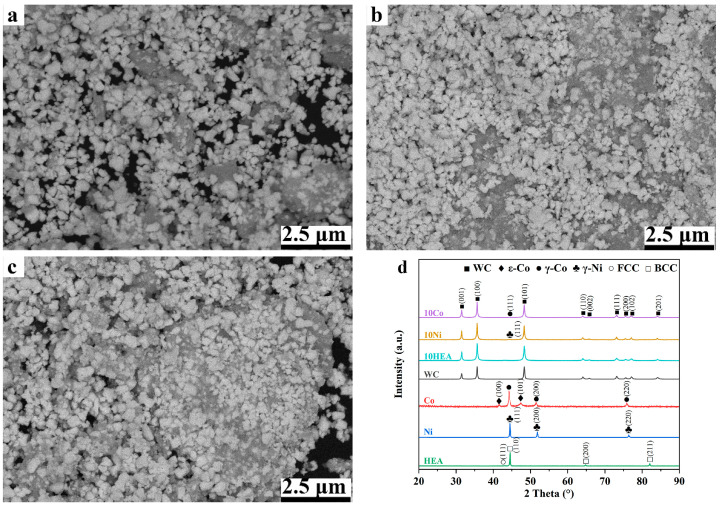
Mixed raw powders of WC-based cermets with different binder phases: (**a**) SEM micrographs of WC-10Co; (**b**) SEM micrographs of WC-10Ni; (**c**) SEM micrographs of WC-10HEA; (**d**) XRD patterns.

To prepare WC-based cermets with a 10 wt.% binder phase, the raw powders were first weighed using a balance with the accuracy of 0.01 mg, and the mass ratio of WC to binder powder was 9:1. Then, the weighed powders were homogeneously dispersed by means of a planetary ball miller (YXQM-2L, MITR, Changsha, China). The weighed powders and cemented carbide balls were mixed into cemented carbide cans. The weight ratio of ball to powder was set as 5:1. The WC/binder powders underwent ball milling for 40 h to ensure adequate mixing and refinement. Milling was carried out using the wet method in an ethanol medium. High-purity argon was introduced into the ball mill to prevent the powder’s oxidation during ball milling. After grinding, the powders were dried in a vacuum rotary evaporator at 50 °C for 3 h to remove ethanol. Figure 2 exhibits the morphology and XRD patterns of the mixed powders after milling. As shown, fine WC particles and the binder phase have been uniformly mixed to each other. From the XRD patterns, it can be found that there’s no mechanical alloying during the mixing process.

### 2.2. Sintering Technique

In this work, the block WC-based cermets were prepared using the spark plasma sintering (SPS) method. First, the mixed powders were placed into the designed graphite mold with a diameter of 30 mm. Graphite heads with the same diameter were used to extend pressure to the powders during the sintering process. Before sintering, the powders were pre-pressed under a pressure of 10 MPa. Subsequently, the pressure was gradually increased to 40 MPa, while the temperature was increased simultaneously. The temperature was raised up to 1300 °C at a heating rate of 50 °C/min. The mold was kept for a duration of 5 min at the highest sintering temperature and pressure condition to ensure the complete densification of the sample. After cooling to room temperature, a WC-based block could be obtained with dimensions of φ 30 mm × 10 mm. In addition, the whole sintering process was conducted in a vacuum with the level of 7 Pa. For brevity, the WC-based samples prepared in this paper were named as WC-10Co, WC-10Ni and WC-10HEA according to the species of binder phase.

### 2.3. Microstructure Observation

To observe the microstructure of the WC-based cermet, the specimens were removed from the sinter blocks by utilizing electric sparking cutting, and the specimens were polished on a diamond disk to obtain a mirror finish. The microstructure was observed by a scanning electron microscope (SEM, SU8230, Hitachi, Tokyo, Japan) equipped with an energy-dispersive X-ray spectroscopy detector (EDS, Oxford Instruments, Abingdon, UK). The phase composition was examined using X-ray diffraction (XRD) on a BRUKER D8 Advance diffractometer (XRD, BRUKER D8 Advance diffractometer, Billerica, MA, USA)with Cu-Kα radiation. The voltage and electricity were set as 40 kV and 30 mA, respectively. The specimen was scanned in the 2θ ranging from 20° to 90° with a step-scan mode (0.02° per step). To quantitatively characterize the size of the WC particles, the average diameter was measured with Image J software (V 1.8.0) using the line intercept method.

### 2.4. Mechanical Properties

**Figure 3:**
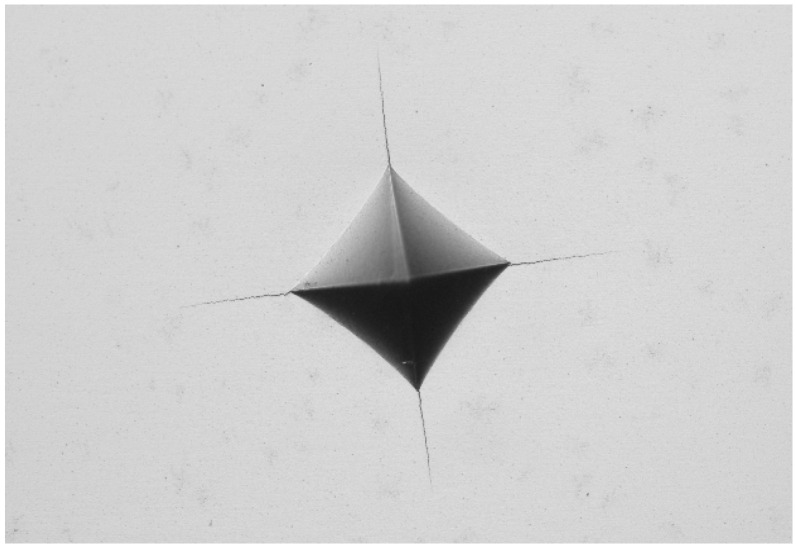
Images of the Vickers indentation with cracks.

The densities of all sintered samples were measured using the Archimedes’ principle method. The hardness was tested on Vickers hardness tester with a load of 30 kg and dwelling time of 15 s. At least 10 positions were randomly tested to acquire the average value of the hardness. A three-point bending test was performed to evaluate the transverse rupture strength (TRS), according to the standard of GB/T 6569-2006 [31]. The specimens for three-point bending test had dimensions of 3 mm × 4 mm × 20 mm [32]. Figure 3 shows the image of Vickers indentation with cracks propagating from the corners. The fracture toughness of the sintered cermets was measured using the indentation method. By measuring the crack length on the corner of the indentation, the fracture toughness can be calculated with the following equation [33]:

(1) $$K_{IC}=0.15\sqrt{\frac{{HV}_{30}}{\sum_{i=1}^{4}L_{i}}}$$

where *K_IC_* is the fracture toughness (MPa·m^1/2^), *HV*_30_ is the Vickers hardness(N/mm^2^) and *L_i_* is the crack length (m).

### 2.5. Wear Test

In this work, dry sliding wear tests on three WC-based cermets were performed using a pin-on-disk rotating tribometer (UMT-TriboLab; Bruker, Billerica, MA, USA) at room temperature. All tested samples were made into pins with a diameter of 6 mm and chamfered. A Si_3_N_4_ disk with a diameter of 45 mm was selected as the counterpart material. The hardness of the Si_3_N_4_ disk was tested to be 20.6 GPa. Before friction and wear tests, the surfaces of the Si_3_N_4_ disks were mechanically polished with 2000 grit sandpaper. Then, the pin specimens and disks were all ultrasonically cleaned in ethanol and then dried thoroughly in hot air. During the wear process, a constant load of 40 N was exerted on the samples and the wear speed was set as 0.2 m/s. For each test, the lasting time of each wear test was 30 min with a wear distance of 360 m. Before and after wear, the specimens were weighed to calculate the wear weight loss. The mass loss was measured with an electronic balance with an accuracy of 0.01 mg. Similarly, three repeated experiments were performed under the same conditions to ensure the repeatability of the results. The specific wear rate of the pin was calculated using Equation (2):

(2) $$W_{R}=\frac{W_{V}}{P\times D_{S}}$$

where *W_R_* is the wear rate (mm^3^/(N·m)), *W_V_* is the volumetric wear of the specimen (mm^3^), *P* is the applied test load (N) and *D_S_* is the sliding distance (m). The features of the wear surfaces were observed using SEM equipped with EDS.

## 3. Results and Discussion

### 3.1. Microstructure of the Sintered WC-Based Cermets

**Figure 4:**
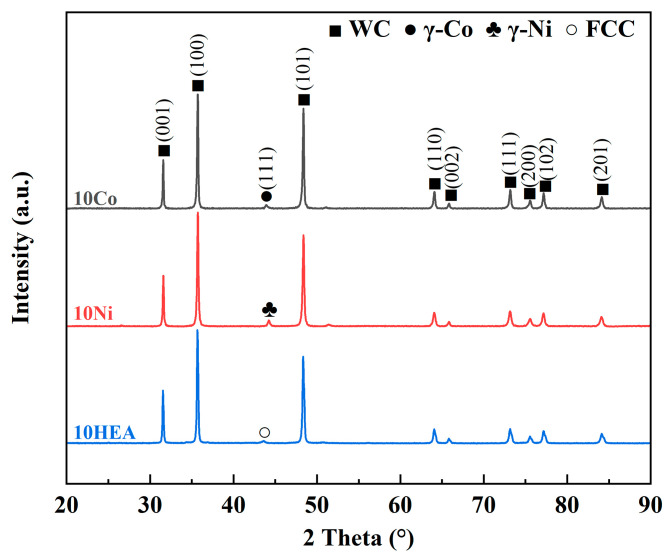
XRD patterns of WC-based cermets with different binder phases.

To identify the phase composition, Figure 4 shows the XRD patterns of WC-based cermets with three kinds of binder phases. It reveals that the as-sintered WC-based cermets are mainly composed of WC and binder phase, which is consistent with the mixed powders before sintering. For WC-10Co, WC-10Ni and WC-10HEA, the binder phases are indicated as Co, Ni and FCC phases, respectively. This result agrees well with that of Luo et al. in which WC-20HEA bulk was also composed of WC and HEA [11]. No other carbides or oxides can be detected for all three sintered samples due to the rapid sintering using the SPS method. On the other hand, this result implies that the as-prepared ultra-fine grain WC-based cermets have good mechanical properties due to the absence of impurities with low strength [9].

**Figure 5:**
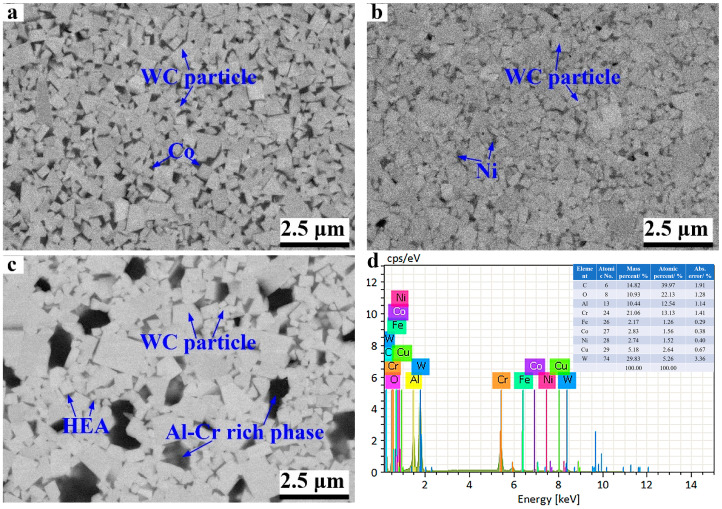
SEM micrographs of WC-based cermets with different binder phases: (**a**) WC-10Co; (**b**) WC-10Ni; (**c**) WC-10HEA; (**d**) EDS analysis of the black phase.

To further observe the microstructure of sintered WC-based cermets, Figure 5 exhibits the SEM micrographs of three samples with different binder phases. As shown, there were no visible micro-voids inside the WC-based cermets prepared in this work, indicating the relatively high degree of densities. By comparison, the size of the WC particles seems to vary with the binder phase. For WC-10Co, the size of WC particles is the finest, while WC-10HEA holds the coarsest WC particles. This indicates that the HEA binder is not conducive to hindering the growth of WC particles, which is a different view to the literature [8,11]. The excessive growth of the WC grain is supposed to be caused by the poor wettability between the WC and HEA phases. The intrinsic reason will be further investigated in the following research. On the other hand, only two kinds of phases, namely WC and Co or Ni, can be observed in the WC-10Co and WC-10Ni samples. However, there is a distinct dark phase in WC-10HEA, except for WC and the HEA binder phase. According to the EDS analysis, the dark phase is rich in Al, Cr and O, implying this phase to be oxides. Zhao et al. [2] reported that the oxidation of Mn and Cr took place during the preparation of coarse WC-10CoCrFeMnNi cemented carbide. The oxides may be detrimental to the sintering ability of the WC-based cermets, which may account for the low relative density. But the hardness is supposed to be improved due to the existence of oxide particles.

**Figure 6:**
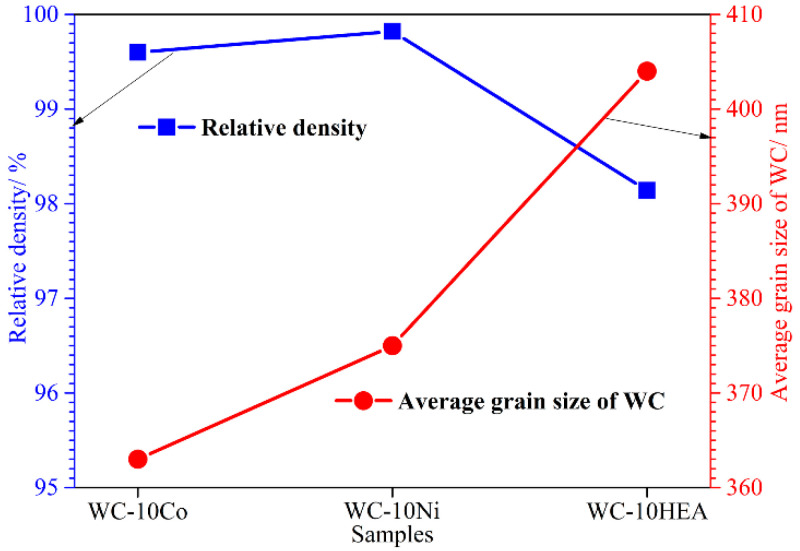
Relative density and average grain size of WC-based cermets.

Furthermore, the average grain size of WC particles was measured, as shown in Figure 6. Firstly, the average grain size is lower than 410 μm for all three cermets, indicating that the sintered samples belong to the ultra-fine grain WC-based cermet [34]. The fine WC size is beneficial in improving the mechanical properties of the cermets [35]. By comparison, the size of WC gradually enlarges when the binder phase varies from Co to Ni and AlCoCrNiFeCu HEA. This phenomenon is exactly consistent with the observation in Figure 5. According to the Hall–Petch equation [36], strength and hardness have a tight relationship with the grain size. Therefore, the relatively higher WC grain size is supposed to be detrimental to the bending strength and hardness of WC-10HEA [37]. In addition, relative density is also a key factor affecting the bending strength of WC-based cermets. Therefore, the relative density for the three cermets were measured, as shown in Figure 6. The relative densities of WC-10Co and WC-10Ni are much higher than that of WC-10HEA. This may be attributed to the worse surface wettability of the AlCoCrNiFeCu alloy [23]. Likewise, the lower density is harmful to the mechanical properties of WC-based cermets [38].

### 3.2. Mechanical Properties

**Figure 7:**
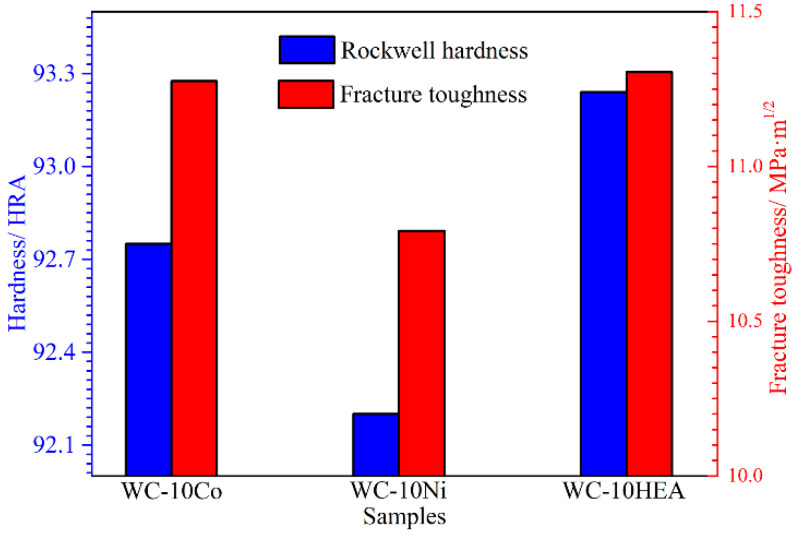
Hardness and fracture toughness of WC-based cermets with different binder phases.

Hardness and toughness are the important factors for the wear performance of samples. Figure 7 shows the hardness and fracture toughness of WC-based cermets with different binder phases. Compared to WC-10Co, the hardness of WC-10HEA can be improved from 92.75 to 93.24 HRA, while the hardness of WC-10Ni decreases to 92.2 HRA. In spite of the lower relative density and larger grain size, WC-10HEA shows the highest hardness. On the one hand, the hardness of AlCoCrNiFeCu HEA is higher than that of Co and Ni [25], which contributes to the higher hardness of WC-10HEA. On the other hand, the appearance of a newly Al-Cr rich phase may also be conducive to improving the hardness. For all three cermets prepared using the SPS method in this work, the Rockwell hardness is basically higher than 92HRA, which is the important factor for guaranteeing superior wear resistance [39,40].

**Figure 8:**
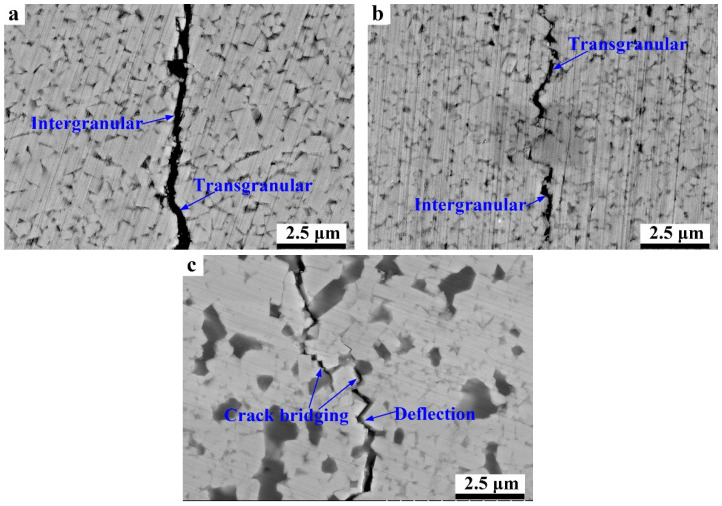
Crack propagation path of WC-based cermets with different binder phases: (**a**) WC-10Co; (**b**) WC-10Ni; (**c**) WC-10HEA.

In addition, WC-10HEA shows the highest fracture toughness of about 11.3 MPa·m^1/2^, as exhibited in Figure 7. By comparison, WC-10HEA possesses both superior hardness and fracture toughness than traditional WC-10Co. Given this result, it can be concluded that WC-10HEA is supposed to have excellent wear resistance. However, the fracture toughness of WC-10Ni is much lower than that of WC-10Co. To further unravel the toughening mechanism, the crack propagation path was observed, as shown in Figure 8. The cracks not only propagate along the grain boundaries of WC but also pass through WC particles. As for WC-10Co and WC-10Ni, it can be observed that the cracks mainly travel along the boundaries of WC particles. Nevertheless, the crack deflection and bridging are very evident for the WC-10HEA cermet. Generally, the cracks’ deflection and bridging are conducive to improving the fracture toughness of ceramics, due to the consumption of energy [32,41]. In WC-10HEA, WC particles are much coarser, so that the crack has to propagate a long distance to bypass them. Furthermore, a crack-bridging phenomenon seems to appear when meeting the Cr-Al rich phase. The two factors are supposed to account for the superior fracture toughness of WC-10HEA.

**Figure 9:**
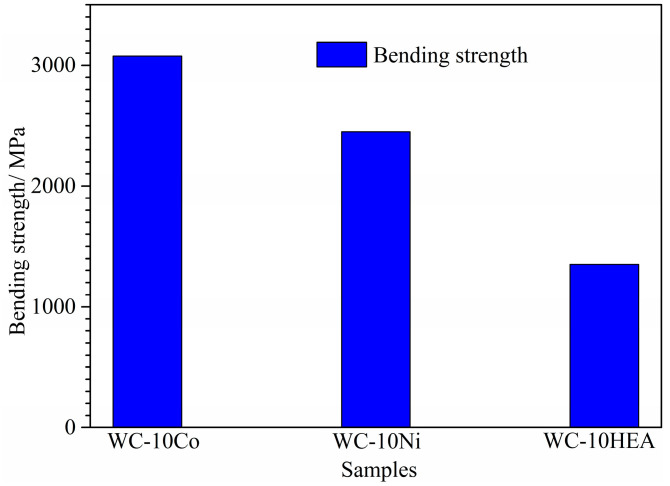
Bending strength of WC-based cermets with different binder phases.

Furthermore, the bending strength of the three WC-based cermets with different binder phases is shown in Figure 9. By comparison, WC-10Co shows the highest bending strength, while WC-10HEA shows the lowest value. This indicates that the substitution of HEA for Co is detrimental to the bending strength of WC-based cermet. The bending strength of WC-10Co reaches 3075 MPa by utilizing the present technique with the SPS method. However, the bending strength of WC-10HEA is only half of that of WC-10Co, with the value of about 1350 MPa. According to the above analysis, the relative density is the lowest and the size of WC particle is the largest, both of which are harmful to the bending strength of WC-10HEA [42]. In addition, the poor wettability may also be an important reason, considering that the pulling out of WC particles is the main fracture mode. Therefore, it is essential to further improve the bending strength of WC-10HEA cermet to extend its industrial applications.

**Figure 10:**
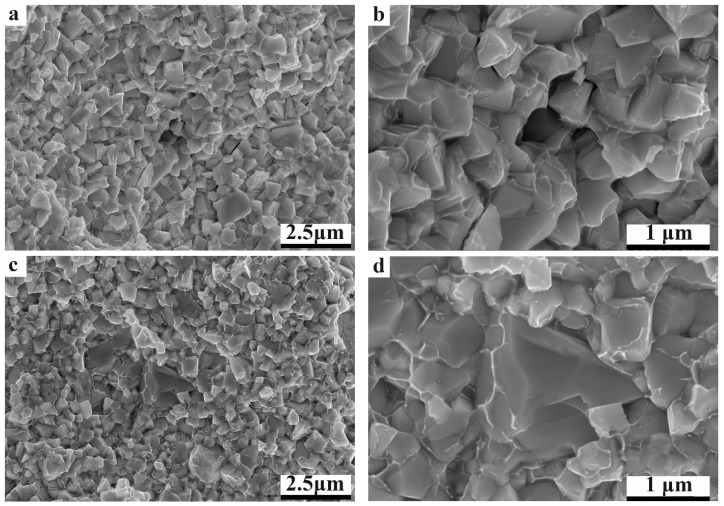

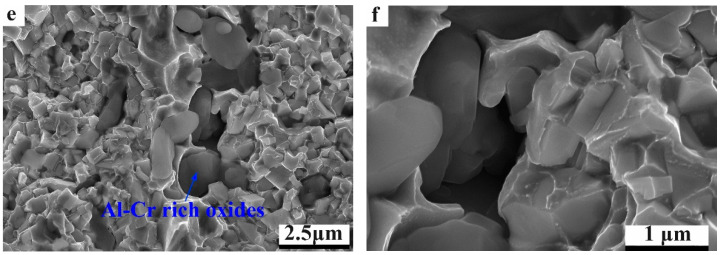
Fracture surfaces of WC-based cermets with different binder phases: (**a**,**b**) WC-10Co; (**c**,**d**) WC-10Ni; (**e**,**f**) WC-10HEA.

To disclose the fracture mechanism during the bending test, the fracture surfaces of all three cermets were observed, as shown in Figure 10. From the fracture surfaces, it can be seen that the WC-10HEA sample has the largest WC grain size. Furthermore, both transgranular and intergranular fractures can be observed on the fracture surfaces of the WC-based cermets. For WC-10Co and WC-10Ni, the fine particles tend to pull out from the binder phase, while the coarse particles tend to fracture during the bending process. The is because the WC phase has a low fracture toughness, so that it is easy to fracture during the bending process [1]. Therefore, the intergranular fracture phenomenon is more common in the WC-10HEA cermet, which is bad for the bending strength. In addition, it can be clearly seen that the black oxides in WC-10HEA have weak bonding to the WC particles. Thus, cracking is supposed to preferentially originate from these sites due to the high tendency of stress concentration. In this case, it is to be expected that the bending strength of WC-10HEA is the poorest, considering the coarsest grain and detrimental oxide phase.

### 3.3. Wear Resistance

**Figure 11:**
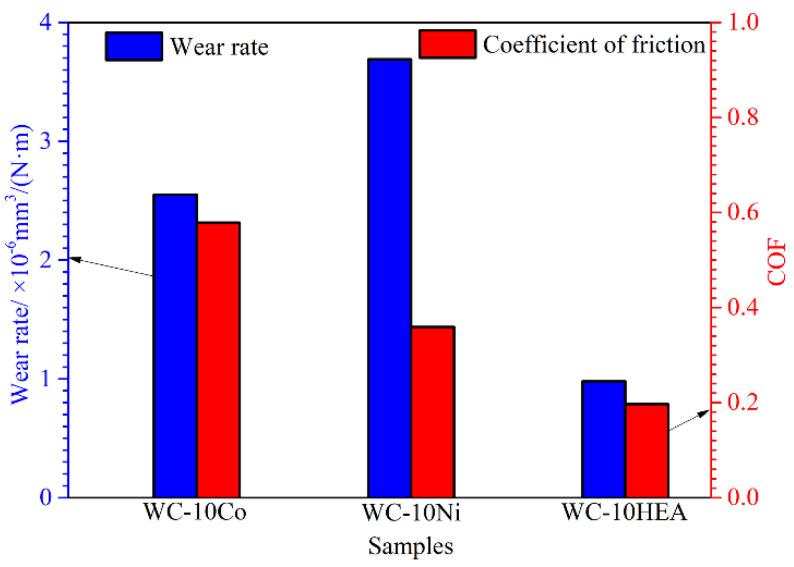
Wear rate and COF of WC-based cermets with different binder phase.

By testing the dry sliding wear properties of the cermets, the wear rate and coefficient of friction (COF) of WC-based cermets can be obtained, as shown in Figure 11. By comparison, WC-10HEA shows the lowest wear rate of 0.98 × 10^−6^ mm^3^/(N·m) and COF of 0.196. Compared to WC-10Co, the wear rate and COF decrease by 45.3 and 65.5% for WC-10HEA, respectively. Likewise, the wear rate and COF of WC-10HEA decrease by about 74.0 and 45.4%, respectively. Thus, it can be concluded that WC-based cermet with AlCoCrNiFeCu HEA binder phase has superior friction and wear properties. Especially, there is a self-lubricating effect for WC-10HEA compared with the traditional WC-based cermets with Co and Ni binder phases [43]. Table 2 lists the comparative results of the microstructure, mechanical properties and wear properties of WC-based cermets with different binder phases. As seen, WC-10HEA possesses the highest hardness and fracture toughness, which is beneficial in improving wear resistance. Simultaneously, AlCoCrNiFeCu HEA binder phase and its oxides during the wear process can play a crucial role in reducing the COF [44]. Consequently, the wear rate of WC-10HEA can be greatly decreased compared with the WC-10Co and WC-10Ni samples. This is important to improve the application properties of WC-based cermets as machining and molding tools.

**Figure 12:**
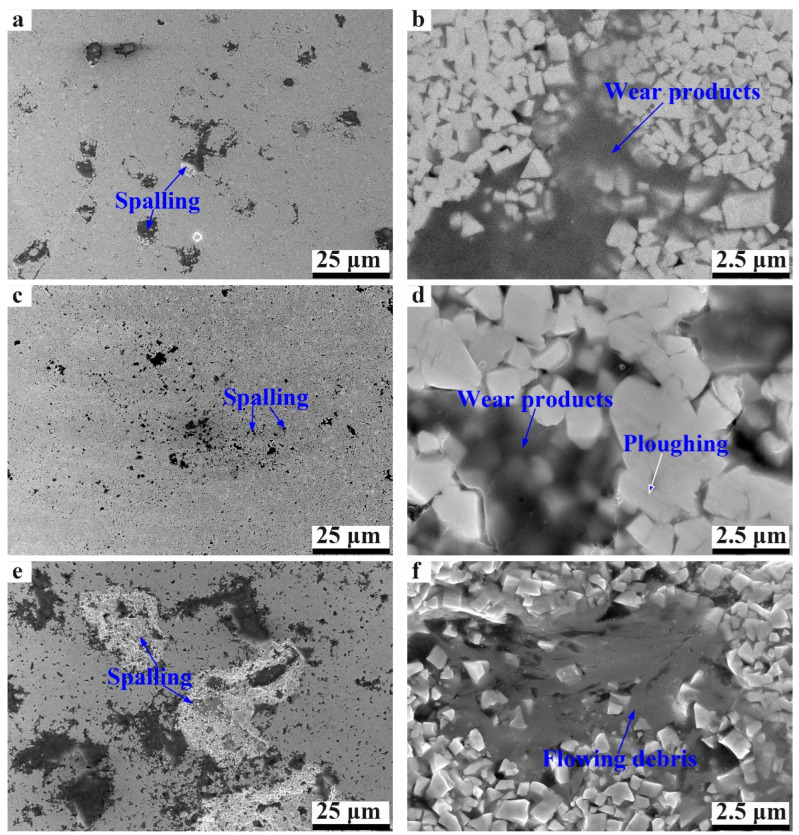

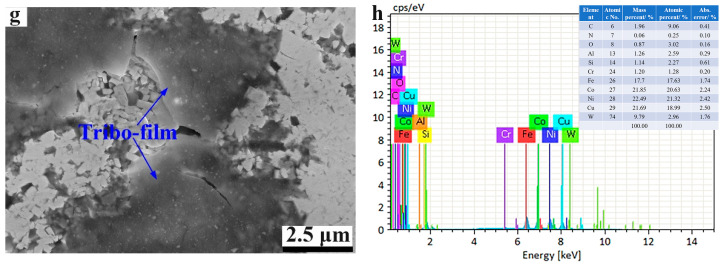
Wear surfaces of WC-based cermets with different binder phases: (**a**,**b**) WC-10Co; (**c**,**d**) WC-10Ni; (**e**–**g**) WC-10HEA; (**h**) EDS analysis of the tribo-film of WC-10HEA.

To unravel the wear mechanisms of different cermets, the wear surfaces were observed for all three cermets, as shown in Figure 12. Ploughing dominates the wear process of WC-based cermets during dry sliding against a Si_3_N_4_ counter, despite the wear tracks not being clear [24,45]. From the magnified morphology in Figure 12d, a few ploughing furrows can be observed, which confirms the main damage form of ploughing. Furthermore, a few peeling pits can be found distributed all over the wear surface of WC-based cermets, which may be the reason for the increase in the COF. It is common knowledge that higher surface roughness contributes to a higher COF. From the magnified micrographs, it can be found that the pits are filled with some dark wear products. According to the EDS analysis in Table 3, the wear products are mainly composed of O, W and Si elements for WC-10Co. Thus, the wear products are supposed to be oxides of wear products due to the thermal effect during the wear process. Generally, the existence of oxides on the wear interface can play a positive role in reducing the COF [45,46,47]. On the other hand, it can be concluded that the cermet and Si_3_N_4_ disk can be simultaneously abraded during the dry sliding process owing to the existence of Si inside the wear products.

For WC-10Ni, the spalling pits seem to be smaller than those of WC-10Co. And the wear products filled in these pits are detected to be composed of O, W, C, Si and Ni. The proportion of W and C is relatively higher than that for WC-10Co, indicating the wear damage of the cermet sample is more serious. This is mainly because of the lower hardness of WC-10Ni. In addition, the smaller spalling pits contribute to the smoother wear surface, so that the COF is lower than that of WC-10Co. Thus, WC-10Ni shows a lower COF than WC-10Co in spite of the higher wear rate. On the other hand, the spalling pits on the wear surface of WC-10HEA are obviously more numerous and larger than those of WC-10Co. This may be attributed to the weak bonding between the WC particles and the HEA binder phase. However, some flat film of wear products can be found to adhere to the wear surface, which is supposed to be beneficial in reducing the COF. Furthermore, the wear debris at some sites shows the feature of flowing for WC-10HEA, which is supposed to play lubricating effect during the wear process. According to the EDS analysis, the flowing regions are rich in Fe, Co, Ni and Cu, and only small amounts of W, C and Si can be detected. Therefore, it can be concluded that the wear debris containing Fe, Co, Ni and Cu shows better lubricating effects, so that the wear damage can be greatly cut down for the WC-10HEA and Si_3_N_4_ counterface. In summary, WC-10HEA has superior anti-wear performance due to its higher hardness and fracture toughness as well as good self-lubricating properties.

## 4. Conclusions

WC-based cermets with the binder phase of Co, Ni and AlCoCrNiFeCu HEA were sintered using the SPS method in this work, and the microstructure, mechanical properties and wear behaviors were systematically investigated to explore the potential of HEA as a binder phase. The following conclusions can be drawn:

(1) WC-based cermets fabricated using SPS show ultrafine WC grains with an average size less than 410 nm. WC-10HEA shows larger WC grain size and lower relative density than WC-10Co and WC-10Ni.
(2) WC-10HEA cermet has the highest hardness and fracture toughness of 93.2 HRA and 11.3 MP·m^1/2^, respectively, owing to the higher hardness of the HEA phase and longer propagation paths during fracturing.
(3) The bending strength is only 1349.62 MPa, which is much lower than that of WC-10Co; the poor bending strength is mainly attributed to the low relative density, coarser WC particles and existence of a Cr-Al rich phase.
(4) Compared to WC-10Co and WC-10Ni, WC-10HEA shows the lowest wear rate of 0.98 × 10^−6^ mm^3^/(N·m) and a COF of 0.196. The higher hardness and fracture toughness contribute to the higher wear resistance, while the wear product film consisting of Fe, Co, Ni and Cu improves the lubricating performance.

## Figures and Tables

**Table 1:** Information on the raw powders.

Species	Purity	D50/μm	Producers
WC	>99.9%	0.6	Ying Rui Technology Co., Ltd., Shenzhen, China
Co	>99.9%	1.0	
Ni	>99.9%	1.0	
HEA	>99.9%	10.0	

**Table 2:** Physical, mechanical and wear properties of WC-based cermets with different binder phases.

Samples	Binder Phase	Grain Size/nm	Density, %	Hardness/HRC	Vickers Hardness/HV	Fracture Toughness/MPa·m^1/2^	Bending Strength/MPa	COF	Wear Rate/10^−6^ mm^3^/(N·m)
WC-10Co	Co	363	99.6	92.75	1911.5	11.27571	3075.78	0.5789	2.55
WC-10Ni	Ni	375	99.82	92.2	1864.8	10.7915	2450.23	0.3593	3.69
WC-10HEA	AlCoCrNiFeCu	404	98.14	93.24	2055.3	11.30462	1349.62	0.1965	0.98

**Table 3:** EDS analysis of the wear surface (wt.%).

Samples	W	C	Si	O	Co	Ni	Al	Cr	Fe	Cu
WC-10Co	19.04	5.64	16.32	54.66	2.13					
WC-10Ni	22.05	12.81	15.41	46.01		3.65				
WC-10HEA	2.96	9.06	2.27	3.02	20.63	21.32	2.59	1.28	17.63	18.99

## Data Availability

Data are contained within the article.

## References

[1] Ghasali E., Ebadzadeh T., Alizadeh M., Razavi M. (2018). Mechanical and microstructural properties of WC-based cermets: A comparative study on the effect of Ni and Mo binder phases. Ceram. Int..

[2] Zhao Z., Wang K., Hu Y., Dong Y. (2023). Microstructure and properties of coarse WC-10CoCrFeMnNi cemented carbide by mechanical alloying and hot pressing sintering. Mater. Today Commun..

[3] Gille G., Szesny B., Dreyer K., van den Berg H., Schmidt J., Gestrich T., Leitner G. (2002). Submicron and ultrafine grained hardmetals for microdrills and metal cutting inserts. Int. J. Refract. Met. Hard Mater..

[4] Tang X., Wang Z., Huang L., Wang X., Chang T., Huang P., Zhu Z. (2023). Preparation, properties and microstructure of high hardness WC-Co cemented carbide tool materials for ultra-precision machining. Int. J. Refract. Met. Hard Mater..

[5] Wu Y., Lu Z., Qin Y., Bao Z., Luo L. (2023). Ultrafine/nano WC-Co cemented carbide: Overview of preparation and key technologies. J. Mater. Res. Technol..

[6] Chang S.-H., Chen S.-L. (2014). Characterization and properties of sintered WC–Co and WC–Ni–Fe hard metal alloys. J. Alloys Compd..

[7] Santos R.F., Rocha A.M.F., Bastos A.C., Cardoso J.P., Rodrigues F., Fernandes C.M., Sacramento J., Ferreira M.G.S., Senos A.M.R., Fonseca C. (2020). Microstructural characterization and corrosion resistance of WC-Ni-Cr-Mo composite—The effect of Mo. Int. J. Refract. Met. Hard Mater..

[8] Zhou P.L., Xiao D.H., Zhou P.F., Yuan T.C. (2018). Microstructure and properties of ultrafine grained AlCrFeCoNi/WC cemented carbides. Ceram. Int..

[9] Rong H., Peng Z., Ren X., Peng Y., Wang C., Fu Z., Qi L., Miao H. (2012). Ultrafine WC–Ni cemented carbides fabricated by spark plasma sintering. Mater. Sci. Eng. A.

[10] Silvestroni L., Gilli N., Migliori A., Sciti D., Watts J., Hilmas G.E., Fahrenholtz W.G. (2020). Binderless WC with high strength and toughness up to 1500 °C. J. Eur. Ceram. Soc..

[11] Luo W., Liu Y., Luo Y., Wu M. (2018). Fabrication and characterization of WC-AlCoCrCuFeNi high-entropy alloy composites by spark plasma sintering. J. Alloys Compd..

[12] Liang F., Du J., Su G., Zhang P., Zhang C. (2023). Investigating the effect of Al, Mo or Mn addition to CoCrFeNi entropy alloys on the interface binding properties of WC/HEA cemented carbides. Mater. Today Commun..

[13] Zeng S., Jin Y., Zhou R., Wu X., Su W., Tang X. (2024). Strengthening and toughening of WC-Ni alloys by adding novel (Ti, Hf, Ta, Nb, Zr)(C, N) high entropy powder. Int. J. Refract. Met. Hard Mater..

[14] Lee J.-H., Oh I.-H., Jang J.-H., Hong S.-K., Park H.-K. (2019). Mechanical properties and microstructural evolution of WC-binderless and WC-Co hard materials by the heat treatment process. J. Alloys Compd..

[15] Tehrani H.M., Shoja-Razavi R., Erfanmanesh M., Hashemi S.H., Barekat M. (2020). Evaluation of the mechanical properties of WC-Ni composite coating on an AISI 321 steel substrate. Opt. Laser Technol..

[16] Su W., Sun Y., Liu J., Feng J., Ruan J. (2015). Effects of Ni on the microstructures and properties of WC–6Co cemented carbides fabricated by WC–6(Co, Ni) composite powders. Ceram. Int..

[17] Huang Z., Ren X., Liu M., Xu C., Zhang X., Guo S., Chen H. (2017). Effect of Cu on the microstructures and properties of WC-6Co cemented carbides fabricated by SPS. Int. J. Refract. Met. Hard Mater..

[18] Eso O., Wang X., Wolf S., Puzz T. (2024). Property correlations of WC-Co with modified binders. Int. J. Refract. Met. Hard Mater..

[19] Pu G., Lin L., Ang R., Zhang K., Liu B., Liu B., Peng T., Liu S., Li Q. (2020). Outstanding radiation tolerance and mechanical behavior in ultra-fine nanocrystalline Al1.5CoCrFeNi high entropy alloy films under He ion irradiation. Appl. Surf. Sci..

[20] George E.P., Curtin W.A., Tasan C.C. (2020). High entropy alloys: A focused review of mechanical properties and deformation mechanisms. Acta Mater..

[21] Holmström E., Lizárraga R., Linder D., Salmasi A., Wang W., Kaplan B., Mao H., Larsson H., Vitos L. (2018). High entropy alloys: Substituting for cobalt in cutting edge technology. Appl. Mater. Today.

[22] Soria-Biurrun T., Navarrete-Cuadrado J., Lozada-Cabezas L., Ibarreta-Lopez F., Martinez-Pampliega R., Sánchez-Moreno J.M. (2022). Microstructure, mechanical properties and fracture behavior of NiCoCrTiAl and FeNiCoCr new alternative binders for WC based hardmetals. Int. J. Refract. Met. Hard Mater..

[23] Mueller-Grunz A., Alveen P., Rassbach S., Useldinger R., Moseley S. (2019). The manufacture and characterization of WC-(Al)CoCrCuFeNi cemented carbides with nominally high entropy alloy binders. Int. J. Refract. Met. Hard Mater..

[24] Zhang P., Chen J., Cheng Q. (2022). Microstructure and sliding wear behavior of (AlCoCrFeNi)_1−x_(WC)_x_. Ceram. Int..

[25] Yang C.-M., Liu X.-B., Liu Y.-F., Zhu Z.-X., Meng Y., Zhou H.-B., Zhang S.-H. (2023). Effect of Cu-doping on tribological properties of laser-cladded FeCoCrNiCux high-entropy alloy coatings. Tribol. Int..

[26] Nayebi B., Asl M.S., Akhlaghi M., Ahmadi Z., Tayebifard S.A., Salahi E., Shokouhimehr M., Mohammadi M. (2021). Spark plasma sintering of TiB2-based ceramics with Ti_3_AlC_2_. Ceram. Int..

[27] Bathula S., Saravanan M., Dhar A. (2012). Nanoindentation and Wear Characteristics of Al 5083/SiCp Nanocomposites Synthesized by High Energy Ball Milling and Spark Plasma Sintering. J. Mater. Sci. Technol..

[28] Buravleva A.A., Fedorets A.N., Vornovskikh A.A., Ognev A.V., Nepomnyushchaya V.A., Sakhnevich V.N., Lembikov A.O., Kornakova Z.E., Kapustina O.V., Tarabanova A.E. (2022). Spark Plasma Sintering of WC-Based 10wt%Co Hard Alloy: A Study of Sintering Kinetics and Solid-Phase Processes. Materials.

[29] Shon I.-J., Kim B.-R., Doh J.-M., Yoon J.-K., Woo K.-D. (2010). Properties and rapid consolidation of ultra-hard tungsten carbide. J. Alloys Compd..

[30] Buravlev I.Y., Shichalin O.O., Papynov E.K., Golub A.V., Gridasova E.A., Buravleva A.A., Yagofarov V.Y., Dvornik M.I., Fedorets A.N., Reva V.P. (2021). WC-5TiC-10Co hard metal alloy fabrication via mechanochemical and SPS techniques. Int. J. Refract. Met. Hard Mater..

[31] (2006). Fine Ceramics (Advanced Ceramics, advanced Technical Ceramics)—Test Method for Flexural Strength of Monolithic Ceramics at Room Temperature.

[32] Jian Y., Qi H., Zhang J., Kong H., Huang Z., Xing J. (2023). Effects of trace Si addition on the microstructure evolution and mechanical properties of MoAlB ceramic. J. Alloys Compd..

[33] Schubert W.D., Neumeister H., Kinger G., Lux B. (1998). Hardness to toughness relationship of fine-grained WC-Co hardmetals. Int. J. Refract. Met. Hard Mater..

[34] García J., Ciprés V.C., Blomqvist A., Kaplan B. (2019). Cemented carbide microstructures: A review. Int. J. Refract. Met. Hard Mater..

[35] Nino A., Izu Y., Sekine T., Sugiyama S., Taimatsu H. (2017). Effects of ZrC and SiC addition on the microstructures and mechanical properties of binderless WC. Int. J. Refract. Met. Hard Mater..

[36] Furushima R., Katou K., Nakao S., Sun Z.M., Shimojima K., Hosokawa H., Matsumoto A. (2014). Relationship between hardness and fracture toughness in WC–FeAl composites fabricated by pulse current sintering technique. Int. J. Refract. Met. Hard Mater..

[37] Lay S., Antoni-Zdziobek A., Pötschke J., Herrmann M. (2020). Microstructural investigations in binderless tungsten carbide with grain growth inhibitors. Int. J. Refract. Met. Hard Mater..

[38] Kim H.-C., Yoon J.-K., Doh J.-M., Ko I.-Y., Shon I.-J. (2006). Rapid sintering process and mechanical properties of binderless ultra fine tungsten carbide. Mater. Sci. Eng. A.

[39] Bartkowska A., Swadźba R., Popławski M., Bartkowski D. (2016). Microstructure, microhardness, phase analysis and chemical composition of laser remelted FeB-Fe2B surface layers produced on Vanadis-6 steel. Opt. Laser Technol..

[40] Bartkowski D., Bartkowska A. (2017). Wear resistance in the soil of Stellite-6/WC coatings produced using laser cladding method. Int. J. Refract. Met. Hard Mater..

[41] Cha S.I., Hong S.H. (2003). Microstructures of binderless tungsten carbides sintered by spark plasma sintering process. Mater. Sci. Eng. A.

[42] Demir B., Ayas E. (2022). Synthesis of Cr2AlB2 MAB phase via Spark Plasma Sintering: Effect of temperature, dwell time, and Al content. Materialia.

[43] Bei G.P., Gauthier-Brunet V., Tromas C., Dubois S., Scattergood R. (2012). Synthesis, Characterization, and Intrinsic Hardness of Layered Nanolaminate Ti_3_AlC_2_ and Ti_3_Al_0.8_Sn_0.2_C_2_ Solid Solution. J. Am. Ceram. Soc..

[44] Aguilar-Hurtado J.Y., Vargas-Uscategui A., Paredes-Gil K., Palma-Hillerns R., Tobar M.J., Amado J.M. (2020). Boron addition in a non-equiatomic Fe_50_Mn_30_Co_10_Cr_10_ alloy manufactured by laser cladding: Microstructure and wear abrasive resistance. Appl. Surf. Sci..

[45] Cao Z., Jian Y., Zhao Z., Xiao P., Xu L., Huang Z. (2023). On the reaction mechanism of Mo2FeB2-based cermets and wear transition induced by self-lubricating tribo-oxide layer. Int. J. Refract. Met. Hard Mater..

[46] Erfanmanesh M., Shoja-Razavi R., Abdollah-Pour H., Mohammadian-Semnani H., Barekat M., Hashemi S.H. (2019). Friction and wear behavior of laser cladded WC-Co and Ni/WC-Co deposits at high temperature. Int. J. Refract. Met. Hard Mater..

[47] Benamor A., Hadji Y., Kota S., Chiker N., Liu L., Haddad A., Sahraoui T., Ying G., Hadji M. (2022). Friction and wear characteristics of the nanolaminated ternary transition metal boride: Mn_2_AlB_2_. Wear.

